# Combination of Talazoparib and Calcitriol Enhanced Anticancer Effect in Triple−Negative Breast Cancer Cell Lines

**DOI:** 10.3390/ph15091075

**Published:** 2022-08-29

**Authors:** Fu Hou Wong, Vijayaraj Kumar Palanirajan, Edmond Siah Chye Ng, Chung Keat Tan, Eugenie Sin Sing Tan, Farahnaz Amini

**Affiliations:** 1 School of Healthy Aging, Aesthetic & Regenerative Medicine, Faculty of Medicine and Health Sciences, UCSI University, Kuala Lumpur 56000, Malaysia; 2 Faculty of Applied Science, UCSI University, Kuala Lumpur 56000, Malaysia; 3 Faculty of Pharmaceutical Sciences, Department of Pharmaceutical Technology, UCSI University, Kuala Lumpur 56000, Malaysia

**Keywords:** anticancer effect, breast cancer, calcitriol, combined therapy, talazoparib, TNBC, triple−negative breast cancer

## Abstract

Monotherapy for triple−negative breast cancer (TNBC) is often ineffective. This study aimed to investigate the effect of calcitriol and talazoparib combination on cell proliferation, migration, apoptosis and cell cycle in TNBC cell lines. Monotherapies and their combination were studied for (i.) antiproliferative effect (using real−time cell analyzer assay), (ii.) cell migration (CIM−Plate assay), and (iii.) apoptosis and cell cycle analysis (flow cytometry) in MDA−MB−468 and BT−20 cell lines. The optimal antiproliferative concentration of talazoparib and calcitriol in BT−20 was 91.6 and 10 µM, respectively, and in MDA−MB−468, it was 1 mM and 10 µM. Combined treatment significantly increased inhibition of cell migration in both cell lines. The combined treatment in BT−20 significantly increased late apoptosis (89.05 vs. control 0.63%) and S and G2/M populations (31.95 and 24.29% vs. control (18.62 and 12.09%)). Combined treatment in MDA−MB−468 significantly increased the S population (45.72%) and decreased G0/G1 (45.86%) vs. the control (26.79 and 59.78%, respectively). In MDA−MB−468, combined treatment significantly increased necrosis, early and late apoptosis (7.13, 33.53 and 47.1% vs. control (1.5, 3.1 and 2.83%, respectively)). Talazoparib and calcitriol combination significantly affected cell proliferation and migration, induction of apoptosis and necrosis in TNBC cell lines. This combination could be useful as a formulation to treat TNBC.

## 1. Introduction

Breast cancer (BC) is the most common type of cancer among women [1]. Molecular profiling of BC have shown subgroups of breast cancer that have different genetic makeups as well as clinical outcomes, calling for the development of new drugs [2,3]. Triple−negative breast cancer (TNBC) is a subtype of advanced and one of the most aggressive types of BC. It is described by the absence of progesterone receptors (PR), estrogen receptors (ER), and human epidermal growth factor receptor 2 (HER2) in the breast tumor [4,5]. TNBC patients have a higher chance of recurrence within three years after diagnosis, and the mortality rates appear to be higher throughout the next five years. TNBC accounted for 10 to 20% of all invasive BC. It has also been associated with a more advanced disease stage, high mitotic indices, higher grade, BC history in the family, and BRCA1 mutations [5]. Currently, standard treatments for BC involve targeted therapy toward receptors such as ER, PR and HER2, making it a less effective treatment option for TNBC patients [6]. Thus, to date, chemotherapy is the most effective systemic therapy for TNBC patients. However, increased metastasis, early recurrence, and poorer outcomes are still prevalent among these patients after chemotherapy [7,8].

By targeting non−overlapping signaling pathways and lowering cross−resistance risks, combination therapies are attractive to overcome drug resistance [9]. Usually, the combined therapy aims to achieve the therapeutic effect in lower doses and less toxicity and to minimize or delay drug resistance. For instance, when methylene blue activated in the presence of laser irradiation (refer here as methylene blue photodynamic treatment or PDT) and doxorubicin (DOX) were combined to treat TNBC [10], the combined therapy using methylene blue–PDT and DOX induced cancer cell death better than DOX alone. Also, using protopanaxatriol (PPT) and ginsenoside Rh2 in the MDA−MB−231 human breast cancer cell line, the combination of PPT and Rh2 had a better inhibitory effect on cell invasion and migration compared to each drug monotherapy [11].

Calcitriol (1,25−dihydroxyvitamin D3) is a vitamin D active metabolite commonly examined in pharmacological doses as an anti−tumor drug [12]. The antitumoral properties are mediated through several vitamin D receptor mechanisms such as the regulation of cell differentiation, growth arrest, invasion, migration, and apoptosis [13]. Calcitriol has shown antiproliferative, pro−differentiative, and pro−apoptotic effects in cancer cells in both in vitro and in vivo models. In opposition, in normal cells, the calcitriol−dependent activation of some kinase cascades and an increase in intracellular calcium may stimulate positive proliferative effects [14].

The most prevalent germline mutations associated with breast cancer are breast and ovarian cancer susceptibility genes 1 and 2 (BRCA1 and BRCA2) [15]. Talazoparib, previously known as BMN 673, is a poly (ADP−ribose) polymerase (PARP) inhibitor that has been approved to treat patients with metastatic BC with germline BRCA mutations [15]. Even talazoparib has been used to treat cancer patients with mutations in BRCA1/2, but recent findings have reported that in some TNBC cell lines, PARP inhibitors are effective in non BRCA1/2 mutation carriers [16]. The side effects caused by talazoparib include fatigue, anemia, diarrhea, nausea, neutropenia and thrombocytopenia [17].

Considering the rise in the incidence of BC, the reported adverse effects for current treatments and fewer options for TNBC patients, the present study was designed to investigate the effect of calcitriol and talazoparib combination on cell proliferation, migration, apoptosis and the cell cycle in TNBC cell lines. It was hypothesized that the combination of talazoparib and calcitriol could improve the anticancer effect when compared to monotherapies. The rationale for combining talazoparib with calcitriol is because calcitriol has been shown to be a potential PARP1 inhibitor, which could counterbalance the side effects of high doses of talazoparib alone [18,19,20,21]. Also, one of the side effects of talazoparib is anemia, whilst evidence shows that calcitriol reduces anemia and the need for erythropoietin therapy [22,23].

## 2. Results

### 2.1. Antiproliferative Effect of Talazoparib, Calcitriol and Their Combination in TNBC Cells

**Figure 1:**
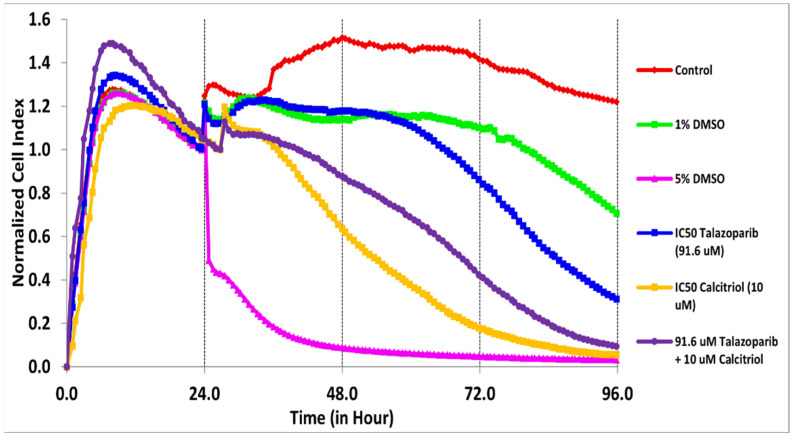
Antiproliferative effect of talazoparib and calcitriol on BT−20. Cells were seeded overnight (1 × 10^4^ cells/well on E−plates) to reach the log phase, then incubated with IC_50_ of 91.6 µM talazoparib and 10 µM calcitriol for 96 h. Data are represented as the mean ± SD (*n* = 3). The drugs were added after 24 h. The green color line is vehicle control (1% DMSO) and the pink color line is the positive control (5% DMSO). Raw data can be found in Supplementary File S1.

**Figure 2:**
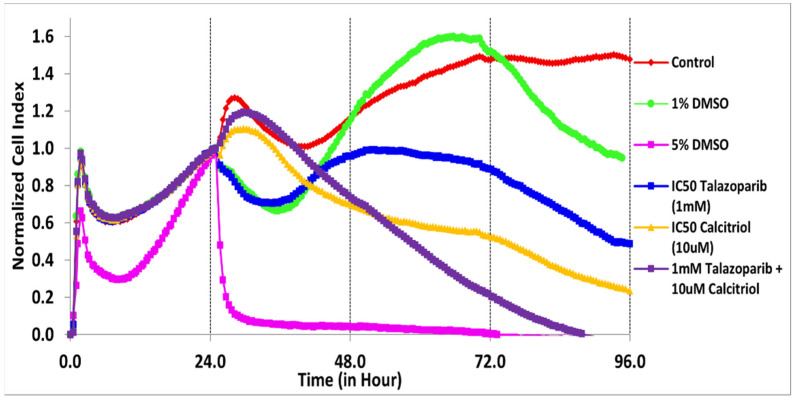
Antiproliferative effect of talazoparib and calcitriol on MDA−MB−468 cells. Cells were seeded overnight to reach the log phase, then incubated with IC_50_ of 1 mM talazoparib and 10 µM calcitriol for 96 h. Data are represented as the mean ± SD (*n* = 3). The drugs were added after 24 h. The green color line is the vehicle control (1% DMSO) and the pink color line is the positive control (5% DMSO). Raw data can be found in Supplementary File S2.

The cytotoxicity of talazoparib, calcitriol and their combination in BT−20 and MDA−MB−468 was monitored for the cell index (CI) values for 96 h (Figure 1 and Figure 2). After treatments, the CI values decreased in a time−dependent manner in both cell lines.

**Figure 3:**
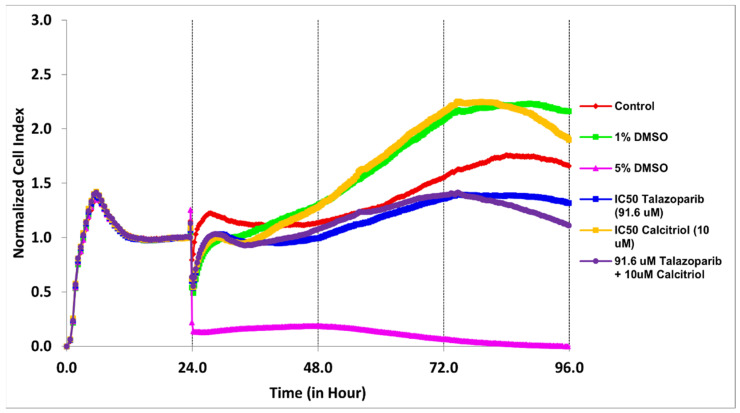
Treatment effects in MRC−5 using BT−20 concentrations. Cells were seeded overnight (1 × 10^4^ cells/well on E−plates) to reach the log phase, then incubated with IC_50_ of 91.6 µM talazoparib and 10 µM calcitriol for 96 h. Data are represented as the mean ± SD (*n* = 3). The drugs were added after 24 h. The green color line is the vehicle control (1% DMSO) and the pink color line is the positive control (5% DMSO). Raw data can be found in Supplementary File S3.

**Figure 4:**
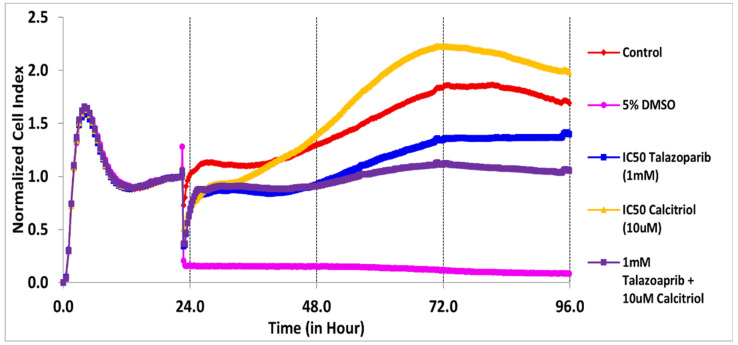
Treatment effects in MRC−5 using MDA−MB−468 concentration. Cells were seeded overnight to reach the log phase, then incubated with 1 mM talazoparib and 10 µM calcitriol for 96 h. Data are represented as the mean ± SD (*n* = 3). The drugs were added after 24 h. The pink color line is the positive control (5% DMSO). Raw data can be found in Supplementary File S4.

In BT−20, the CI values dropped to half of the total cells after being treated for 61 and 28 h with the concentration of 91.6 µM talazoparib and 10 µM calcitriol, respectively (Figure 1 and Table 1). In MDA−MB−468, the CI values dropped to half of the total cells after being treated for 69 and 50 h with the concentration of 1 mM talazoparib and 10 µM calcitriol, respectively (Figure 2 and Table 2). All the treatments in BT−20 and MDA−MB−468 were tested in MRC−5. Results showed that all the treatments up to 96 h have no antiproliferative effect in MRC−5 (Figure 3 and Figure 4), indicating that these treatments targeted cancer cells and not normal cell lines.

### 2.2. Cell Migration Profile of TNBC Cells Treated by Talazoparib, Calcitriol and Their Combination

**Figure 5:**
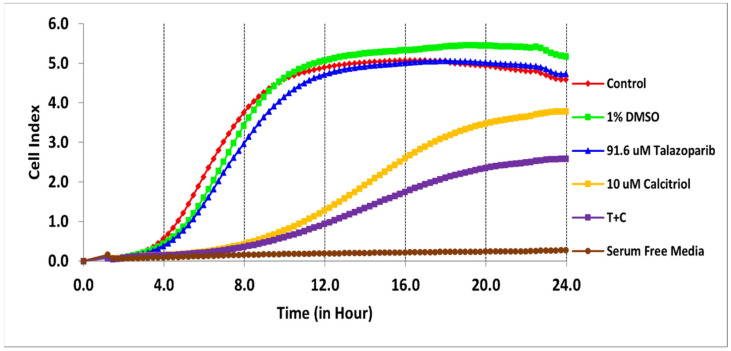
BT−20 cell migration profiles. The experiment was performed in triplicate for each treatment. Real−time migration of BT−20 cells, the slope represents the degree of cell migration (cell index) over time. Data are represented as the mean ± SD (*n* = 3). The red color line is untreated control. The green color line is vehicle control (1% DMSO), and brown color line is negative control (serum−free media). Raw data and details of the statistical analysis can be found in Supplementary File S5 and Table S1, respectively.

The migration profile in the BT−20 cell line was studied (Figure 5). Serum−free media served as the negative control (no cell migration was observed). Calcitriol showed a lower migration rate at 8.3% lower than the untreated control (Figure 5). The combination of 91.6 µM talazoparib and 10 µM calcitriol significantly inhibited migration (39%) compared with the untreated control (*p <* 0.001). Additionally, the combined treatment significantly reduced the migration when compared with talazoparib (*p <* 0.001) and calcitriol monotherapy (*p <* 0.001) after 24 h.

**Figure 6:**
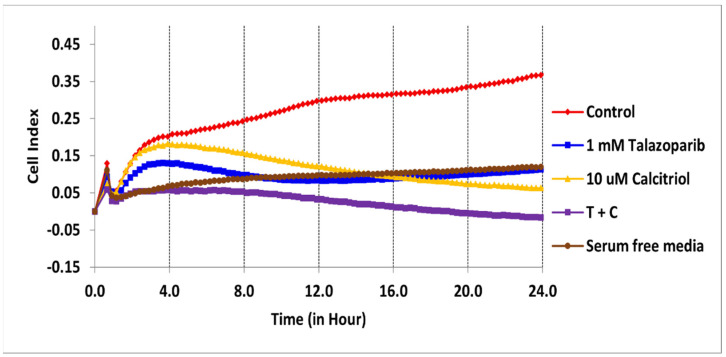
MDA−MB−468 cell migration profiles. The experiment was performed in triplicate for each treatment. Real−time migration of MDA−MB−468 cells, the slope represents the degree of cell migration (cell index) over time. Data are represented as the mean ± SD (*n* = 3). The red color line is the untreated control. The brown color line is the negative control (serum−free media). Raw data and details of the statistical analysis can be found in Supplementary File S6 and Table S2, respectively.

Figure 6 shows the migration profile of the MDA−MB−468 cell line. All treatments significantly decreased the migration rate (*p* < 0.001).

For the statistical analysis, data were compared in terms of the migration rate between the untreated control and the treatment groups by a one−way ANOVA post−hoc test (Tukey) using SPSS (For details of statistical analysis, refer to Supplementary Files S5 and S6).

### 2.3. Talazoparib and Calcitriol Induced Apoptosis in BT−20 Cells

**Figure 7:**
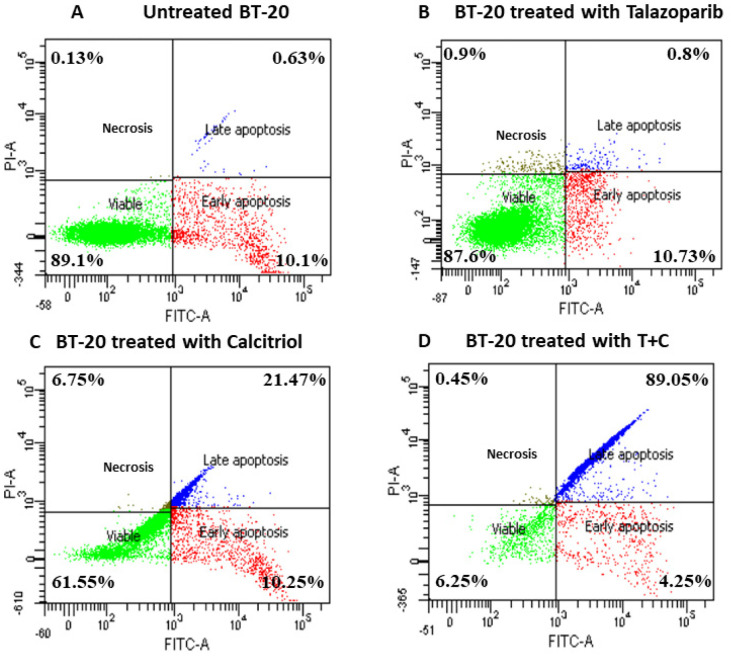
Annexin V/FITC−PI flow cytometry analysis of BT−20. Cells (1 × 10^6^ cells/mL) treated with talazoparib (Panel **B**), calcitriol (Panel **C**), and combined treatment (Panel **D**) for 24 h vs. untreated control (Panel **A**). Cells were dual−stained with Annexin V−FITC and propidium iodide, and the dot plot of BT−20 with different treatments was generated. Each data set is a representative plot of three independent experiments (green dots represent viable cells, red dots represent early apoptosis, blue dots represent late apoptosis and brown dots represent necrosis), while percentages are the mean value of three independent experiments. Raw data can be found in Supplementary File S7.

**Figure 8:**
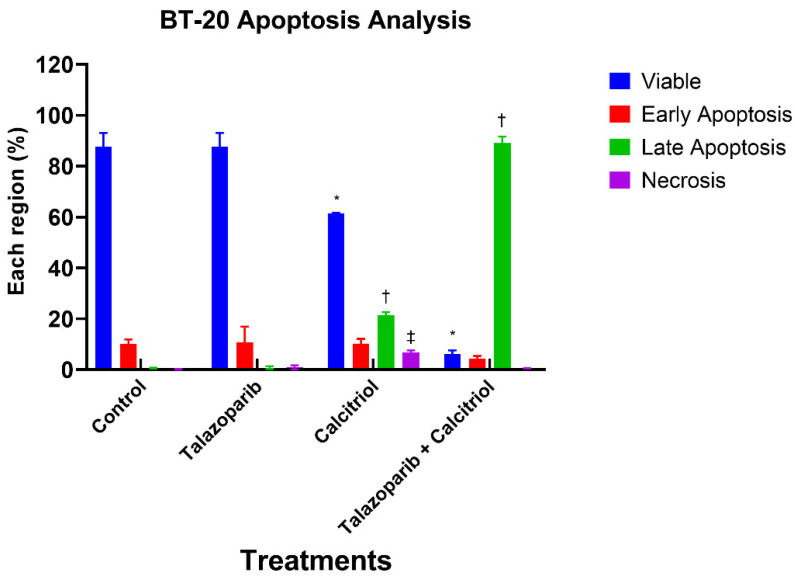
Histogram for apoptosis rate in BT−20. Data are represented as means ± standard deviation (SD) of three independent experiments where (*) indicates a significant difference of viable cells between treatment groups versus the control group as * *p* < 0.05. (†) indicates a significant difference of late apoptosis between treatment groups versus the control group as † *p* < 0.05. (‡) indicates a significant difference of necrosis between treatments versus the control group as ‡ *p* < 0.05. Data were compared between the untreated control and the treatment groups by a one−way ANOVA post−hoc test (Tukey) using SPSS. Details of the statistical analysis can be found in Supplementary Table S3.

An apoptosis assay was performed to determine talazoparib, calcitriol and their combination’s effect on BT−20 death rate after 24 h treatment. For all the treatments, the early apoptosis rates were not significantly different when compared to the control (untreated cells) (the rate of early apoptosis in talazoparib, calcitriol, and their combination were 10.73 ± 6.2%, 10.25 ± 1.9% and 4.25 ± 1.2%, respectively, when compared with the rate of 10.1 ± 1.8% in the untreated control). In the calcitriol−treated group, late apoptosis (*p* < 0.001) and necrosis (*p* < 0.001) were significantly higher when compared to the untreated control. In the combined treatment, only late apoptosis (*p* < 0.001) was significantly higher compared to the untreated control (The rate of late apoptosis in the calcitriol and combined treatment significantly increased by 21.47 ± 1.2% and 89.05 ± 2.6%, respectively) (Figure 7 and Figure 8).

### 2.4. Talazoparib and Calcitriol Induced Apoptosis in MDA−MB−468 Cells

**Figure 9:**
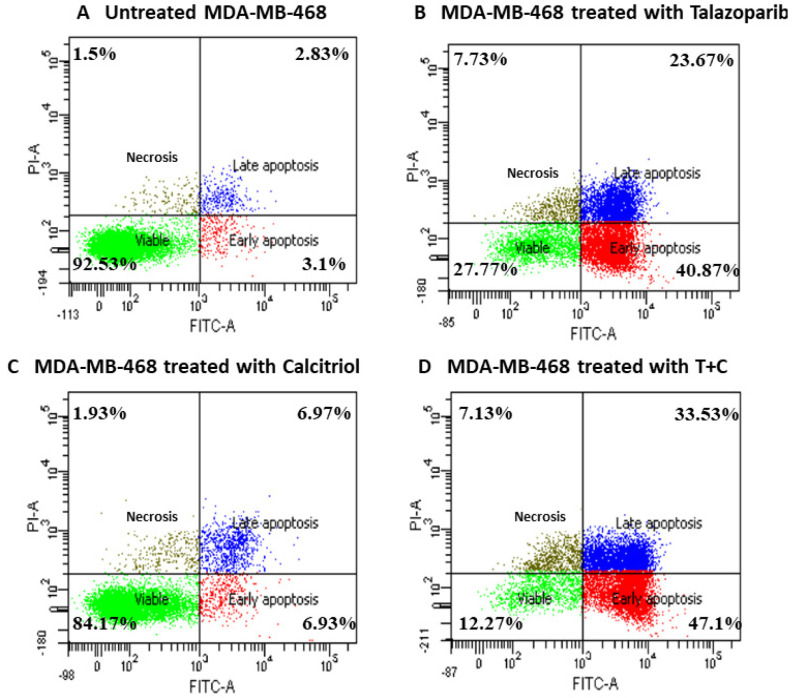
Annexin V/FITC−PI flow cytometry analysis of MDA−MB−468. Cells (1 × 10^6^ cells/mL) were treated with talazoparib, calcitriol, and a combined treatment for 72 h compared to an untreated control. Cells were dual−stained with Annexin V−FITC and propidium iodide. (**A**–**D**) are representative of dot plots and of different treatments in MDA−MB−468. Each data set is representative of three independent experiments, while percentages are the mean value of three independent experiments. Raw data can be found in Supplementary File S8.

**Figure 10:**
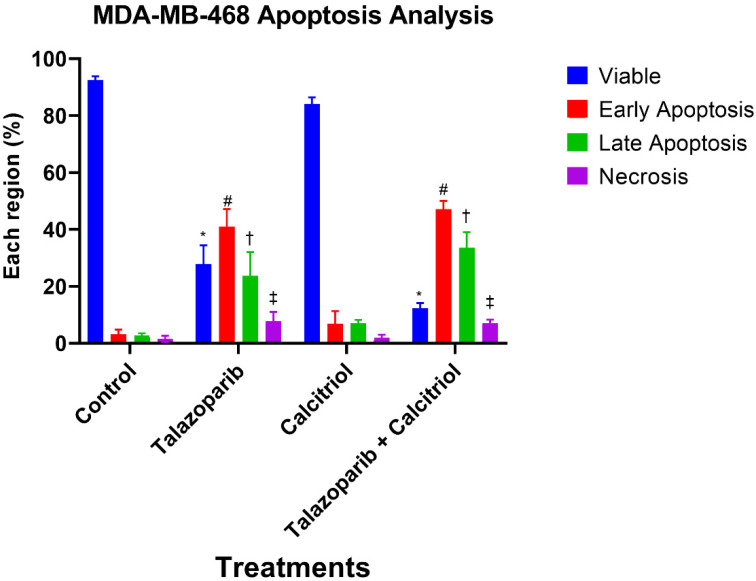
Histogram for apoptosis rate in MDA−MB−468. Data are represented as means ± SD of three independent experiments where (*) indicates a significant difference of viable cells between treatments versus the control group as * *p* < 0.05 vs. untreated control. (#) indicates a significant difference of early apoptosis between treatments versus the control group as # *p* < 0.05. (†) indicates a significant difference of late apoptosis between treatments versus the control group as † *p* < 0.05. (‡) indicates a significant difference of necrosis between treatments versus control group as ‡ *p* < 0.05. Data were compared between the untreated control and the treatment groups by a one−way ANOVA post−hoc test (Tukey) using SPSS. Details of statistical analysis can be found in Table S4.

An apoptosis assay was performed to determine talazoparib, calcitriol and their combination’s effect on MDA−MB−468 death rate after 72 h of treatment. The results of Annexin V−FITC/PI dual staining demonstrated that the early apoptosis and late apoptosis rate in MDA−MB−468 significantly increased in talazoparib and the combined treated groups when compared with the control. In the talazoparib treated group, early apoptosis (*p* < 0.001), late apoptosis (*p* = 0.003) and necrosis (*p* = 0.015) were significantly higher when compared to the untreated control. In the combined treatment, early apoptosis (*p* < 0.001), late apoptosis (*p <* 0.001) and necrosis (*p* = 0.027) were significantly higher when compared with the untreated control group, whilst calcitriol did not statistically affect the necrosis (Talazoparib and combined treatment induced early apoptosis at a rate of 40.87 ± 6.3% and 47.1 ± 2.9%, respectively, vs. 3.1 ± 1.7% in the untreated control group. The rate of late apoptosis in control was 2.83 ± 0.7%, while talazoparib and combined treatment induced 23.67 ± 8.4% and 33.53 ± 5.5%, respectively. Early and late apoptosis in the calcitriol treatment group was 6.93 ± 4.4% and 6.97 ± 1.3%, respectively. Rate of necrosis was 1.5 ± 1.1% in untreated control compared to 7.73 ± 3.3% and 7.13 ± 1.2% in the talazoparib and combined treatment, respectively) (Figure 9 and Figure 10). In comparison, the combined treatment showed significant differences compared with calcitriol in early apoptosis (*p <* 0.001), late apoptosis (*p* = 0.001) and necrosis (*p* = 0.041).

### 2.5. Cell Cycle Arrest in BT−20 Cells

**Figure 11:**
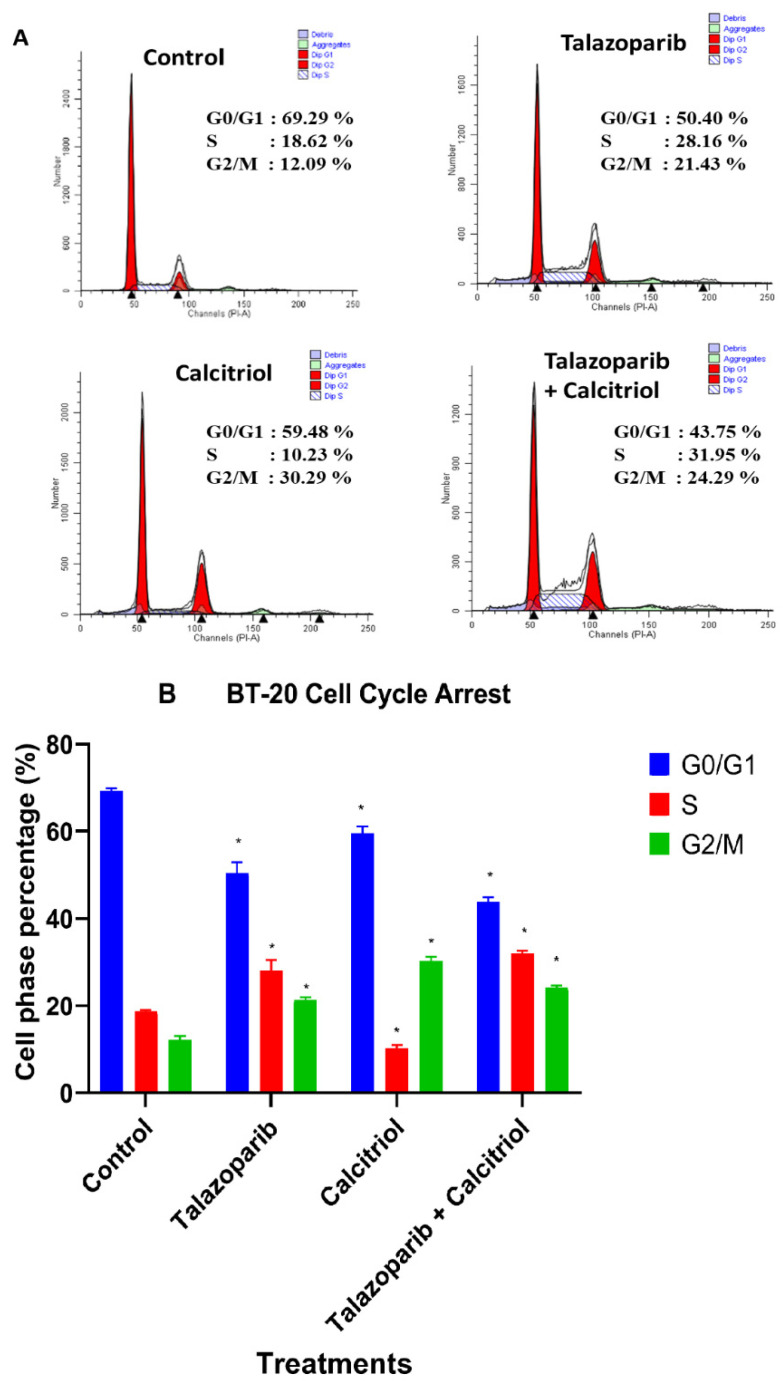
Effect of talazoparib and calcitriol on cell cycle progression in BT−20 cells. (**A**) FACS analysis (Results are representative of three independent experiments). (**B**) Analysis of the cell cycle with talazoparib, calcitriol and combined treatment. BT−20 was treated with 91.6µM talazoparib and 10µM calcitriol for 24 h. In talazoparib and combined treatment, an increase in the populations of G2/M and S phase cells were observed, whereas the population of cells in G0/G1 phase decreased compared to the untreated cells. In calcitriol treatment, an increase in the populations of G2/M phase cells was observed whereas the population of S and G0/G1 phases decreased. The results represent the mean ± SD of three independent experiments. * p < 0.05 vs. untreated control. Data were compared between the untreated control and the treatment groups by one−way ANOVA Post Hoc test (Tukey) using SPSS. Raw data and details of statistical analysis can be found in Supplementary S9 and Table S5, respectively.

Cell cycle analysis was performed to investigate the effect of the talazoparib and calcitriol treatment and their combination on the cell cycle phase distribution of BT−20. Following 24 h of treatment with talazoparib, the S (*p* < 0.001) and G_2_/M (*p* < 0.001) populations of the BT−20 were significantly higher compared to the untreated control. In the calcitriol−treated group, the G_2_/M (*p* < 0.001) population of the BT−20 significantly increased compared to untreated control (talazoparib−induced S and G_2_/M population of the BT−20 was 28.16 ± 2.37% and 21.43 ± 0.58%, respectively vs. untreated control (18.62 ± 0.36% and 12.1% ± 0.91%). For the calcitriol treatment, the G_2_/M population of the BT−20 was 30.29 ± 0.99%. For combined treatment, S and G_2_/M populations of the BT−20 were 31.95 ± 0.7% and 24.29 ± 0.42%, respectively) (Figure 11). In comparison, the combined treatment showed significant differences when compared with talazoparib (*p* = 0.004) and calcitriol in the G_2_/M phase (*p* < 0.001).

### 2.6. Cell−Cycle Arrest in MDA−MB−468 Cells

**Figure 12:**
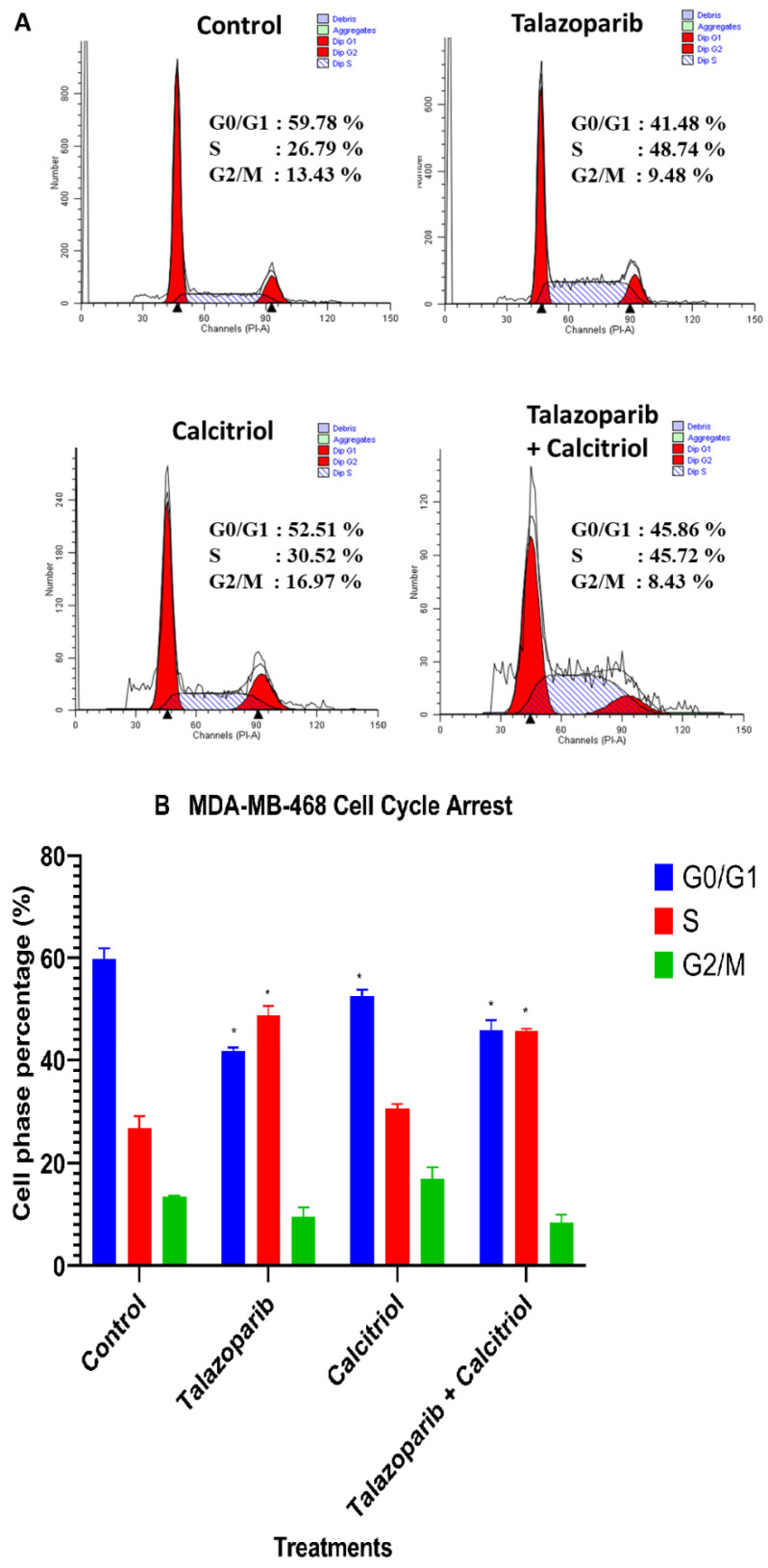
Effect of talazoparib and calcitriol on cell cycle progression in MDA−MB−468 cells. (**A**) FACS analysis (Results are representative of three independent experiments). (**B**) Analysis of the cell cycle with talazoparib, calcitriol and combined treatment. MDA−MB−468 was treated with 1 mM talazoparib and 10 µM calcitriol for 24 h. Compared to the untreated cells, an increase in the population of S phase cells was observed, whereas the population of cells in G0/G1 phase decreased. The results represent the mean ± SD of three independent experiments. * p < 0.05 vs. control. Data were compared between the untreated control and the treatment groups by a one−way ANOVA post−hoc test (Tukey) using SPSS. Raw data and details of statistical analysis can be found in Supplementary File S10 and Table S6, respectively.

Cell−cycle analysis was performed to investigate the effect of the talazoparib, calcitriol and their combination on cell cycle phase distribution in MDA−MB−468. Following 24−h treatment of talazoparib (*p < 0.001*) and combined treatment (*p <* 0.001), S phase population of the MDA−MB−468 significantly increased compared to the untreated control. In addition, G_0_/G_1_ population of the MDA−MB−468 significantly decreased after talazoparib (*p* < 0.001), calcitriol (*p* = 0.004) and combined treatment (*p* < 0.001) compared with untreated control (Talazoparib and combined treatment induced S phase population in MDA−MB−468, (48.74% ± 1.87% and 45.72% ± 0.31%, respectively) compared to the untreated control (26.79% ± 1.61%). In addition, G_0_/G_1_ population of the MDA−MB−468 were 41.78% ± 0.67% in talazoparib, 52.51% ± 1.26% in calcitriol and 45.86% ± 1.39% in combined treatment compared with untreated control (59.78% ± 1.46) (Figure 12). In comparison, the combined treatment showed significant differences compared with calcitriol in the G_2_/M phase (*p* = 0.005).

## 3. Discussion

Combined therapy is promising in treating cancers, especially when lower dosages of drugs are used, which in turn minimizes the side effects and cytotoxicity of long exposure of healthy tissues while still achieving the desired therapeutic results. Among PARP inhibitors, talazoparib has been reported to have the highest efficacy, which is 100−fold more potent than Olaparib [15]. A recent clinical study reported that 39% of breast cancer patients treated with talazoparib had developed anemia [24]. Another study also mentioned that talazoparib had a higher rate of alopecia and anemia [25]. This study hypothesized that the combination of talazoparib and calcitriol could improve the antiproliferation effect when compared to monotherapies. The rationale for the combination of talazoparib with calcitriol is based on the fact that calcitriol has been shown to be a potential PARP1 inhibitor, which could counterbalance the side effects of a high dose of talazoparib alone [18,19,20,21]. Additionally, one of the side effects of talazoparib is anemia, whilst evidence shows that calcitriol improves anemia and lessens the requirement for erythropoietin therapy [22,23]. Furthermore, targeting DNA repair mechanisms as one of the major contributors to cancer using PARP inhibitors seems promising for TNBC patients regardless of their BRCA mutations [16]. The combination of talazoparib with other drugs has been tested to treat different cancers. Children and adolescents have tolerated talazoparib in combination with temozolomide with refractory/recurrent solid tumors, including Ewing sarcoma [26]. Also, a combination of Palbociclib and talazoparib was proposed as a potential treatment for bladder cancer [27]. In seven TNBC cell lines, the combination of carboplatin and talazoparib showed synergistic effects [28]. However, to the best of our knowledge, this is the first combined therapy of talazoparib with calcitriol in treating TNBC cell lines. The synergistic effects of calcitriol with other drugs in their lower concentrations have been reported in previous studies. A lower dose of doxorubicin and genistein were needed to see growth inhibition in breast adenocarcinomas (MCF−7) and prostate carcinomas (LNCaP) when they were combined with calcitriol at a synergistic concentration [29]. Moreover, another study on the human pancreatic cancer model system Capan−1 showed a synergistic effect of calcitriol and gemcitabine when treated over a wide range of concentrations, in turn enhancing the inhibition of cell proliferation [30].

In the present study, both calcitriol and talazoparib monotherapy inhibited cell proliferation in MDA−MB−468 and BT−20. Calcitriol has been previously used both as monotherapy and combined therapy to treat TNBC cell lines [31,32]. In monotherapy, it inhibited TNBC proliferation through a mechanism involving the proinflammatory cytokines IL−1 β and TNF−α. The combination of calcitriol and celecoxib in two breast cancer cell lines showed a cooperative growth−inhibiting effect [33]. Additionally, calcitriol significantly inhibited the proliferation of SUM−229PE (a TNBC cell line) and MCF7 (ER−positive breast cancer cell) [31,32]. Additionally, the combined therapy of calcitriol and TNF−α had a greater cell growth inhibitory effect when compared to monotherapies in breast cancer cells [32]. The combination of calcitriol and menadione (a glutathione−depleting compound) also reduced tumor growth by improving the antiproliferative effect [34]. Furthermore, co−administration of calcitriol with curcumin or resveratrol significantly reduced the cell proliferation of the MBCDF−T cells (a TNBC), which were xenografted in nude mice [12]. However, in normal endothelial cells (EA.hy926 cells), the cell proliferation increased after calcitriol treatment [12]. In MDA−MB−231 cells (a TNBC cell), combined therapy of calcitriol with tyrosine kinase inhibitors notably inhibited cell growth [27]. Overall, calcitriol is a natural vitamin D receptor (VDR) agonist; hence, it can reduce cell viability in breast cancer cell lines that are VDR−positive [35]. In this study, analyzing the cell viability in two TNBC cell lines showed that IC_50_ values of talazoparib in BT−20 cells were 11−fold lower than the IC_50_ in MDA−MB−468. Evaluating the reduction in cell viability and the respective IC_50_ after treatment with talazoparib monotherapy in various cancer cell lines, including breast cancer, has shown different efficiency, likely due to the genetic and epigenetic diversity among these cell lines [27,36].

Cell migration is a crucial step in cancer cell metastasis [37]. The present study observed that the combined treatment has a better anti−migration effect than monotherapies. Treatment with talazoparib for 24 h did not reduce the cell migration in BT−20. Calcitriol reduced cell migration in BT−20 when compared to untreated cells. The combination of talazoparib and calcitriol demonstrated a greater migration inhibitory effect (39%) than calcitriol monotherapy (8%) in this cell line. Similar results were observed from a study on human prostate cancer cell lines, PC−3 and DU145, where cell migration decreased and was inhibited after treatment with calcitriol [38]. Moreover, colon cancer cells (DLD−1 and HCT116) treated with calcitriol for 24 and 48 h showed a reduction in cellular migration by 62 and 80%, respectively [39]. A combination treatment of talazoparib and bazidoxifene on human ovarian cancer cells, SKOV3, has shown a greater inhibitory effect on cell migration than monotherapies [37]. In the present study, for MDA−MB−468, cell migration decreased in both mono− and combined therapy. The synergistic antitumorigenic activity of calcitriol with curcumin in MBCDF−T cell (a breast cancer cell line) also reported that all treatments resulted in a slower migration than vehicle−treated cells. However, when calcitriol and curcumin were combined, the reduction was seen to a greater extent than the monotherapies [12].

In the present study, calcitriol did not significantly increase the apoptosis in MDA−MB−468 cells, which is in agreement with some studies that also found no effect of calcitriol on apoptosis in human lung cancer, malignant pleural mesothelioma, and adrenocortical carcinoma cell lines [40,41,42]. These findings suggest that the antitumor effects of calcitriol in some TNBC cells involve cell cycle arrest and the inhibition of cell cycle progression [40]. Furthermore, the apoptosis analysis in the present study showed that the combined therapy of calcitriol and talazoparib on BT−20 and MDA−MB−468 increased the apoptotic cells. Similar results were reported when the percentage of apoptotic cells increased in talazoparib−loaded nanoemulsion−treated Adriamycin−resistant ovarian cancer cells (NCI/ADR−RES) [43]. However, in another study, the combination of talazoparib and Palbociclib did not increase the apoptosis in bladder cancer cell lines [27].

In the calcitriol−treated BT−20, cells were arrested at G2/M phase, whereas cells treated with the combination of talazoparib and calcitriol were mostly arrested in the S phase. Another study has reported that calcitriol arrested MBCDF−T cells in the G1−phase, whilst calcitriol combined with curcumin arrested cells in S−phase [12]. In the present study, talazoparib and calcitriol treatment increased G2/M arrest in BT−20. Similarly, a study also reported G2/M arrest in HCC1937 (a BRCA1 mutant) and MDA−MB−231 (a BRCA1 wild−type) TNBC cell lines after treatment with talazoparib [44]. Furthermore, co−administration of olaparib (a PARP inhibitor) with suberoylanilide hydroxamic acid in several TNBC cell lines resulted in a higher percentage of cell cycle arrest at the G2/M phase [45]. Additionally, a significant rise in the G2/M population was observed upon treatment with talazoparib in melanoma cells and *Schlafen 11*−deleted cancer cells [46,47]. In MDA−MB−468, the talazoparib and the combined treatment significantly increased the S phase, whereas calcitriol slightly increased in the S and G2/M phase with no significant difference. The higher concentration of talazoparib, which was needed for IC_50_ in MDA−MD−468, may cause a higher portion of cells to be arrested in the S phase. A previous study has reported that among PARP inhibitors, talazoparib treatment created a higher percentage of cells in the S−phase [48].

Talazoparib has shown a diverse level of antiproliferative effects in different cell lines, indicating the impact of varying genetic backgrounds [49]. Similarly, in the present study, MBA−MD−468 was less sensitive to talazoparib when compared to BT−20 as indicated by significant differences in the IC_50_ of 1 mM vs. 91.6 µM. Additionally, a recent study, which has tested the sensitivity of a panel of breast cancer cell lines to metformin, has reported that the cell lines’ sensitivity varied greatly, as seen by variances in IC_50_ that ranged from 0.83 to 10.13 mM [50]. There is no previous study on the combination of talazoparib and calcitriol with antagonist effects, but a study has reported that the mild antagonistic effects of piperaquine, pyronaridine, and naphthoquine may not cause any significant short−term clinical effect in treating malaria [51]. Then, it is essential to investigate the clinical benefit of our findings in pre−clinical studies.

## 4. Materials and Methods

### 4.1. Reagents and Materials

The talazoparib (BMN 673) was purchased from Selleckchem (Houston, TX, USA) (catalogue number S7048−10). Calcitriol was purchased from Tokyo Chemical Inc (Japan) (catalogue number C3078). The stock solution was prepared in dimethyl sulfoxide (DMSO; Nacalai Tesque Inc, Kyoto, Japan) at a 400 and 40 mM concentration for talazoparib and calcitriol, respectively, and stored at −20 °C.

### 4.2. Cell Lines and Cell Culture

All cell lines were purchased from American Type Culture Collection (ATCC: Manassas, VA, USA). Culture media were obtained from ATCC. BT−20 is a triple−negative breast cancer cell line developed in 1958 by Lasfargues and Ozzello from a 74−year−old human female. BT−20 was cultured in Eagle’s Minimum Essential Medium (EMEM) supplemented with 10% fetal bovine serum (FBS) and 100 U penicillin / 0.1 mg/mL streptomycin. The MDA−MB−468 cell line was originally obtained from a pleural effusion of a 51−year−old Black female patient with metastatic adenocarcinoma of the breast in 1977. MDA−MB−468 (TNBC cell line) was cultured in Leibovitz’s L−15 Medium supplemented with 10% FBS and 100 U penicillin / 0.1 mg/mL streptomycin. All cell lines were grown and maintained at 37 °C in a humidified atmosphere (90% relative humidity) with 5% CO_2_.

MRC−5, a normal fibroblast cell line developed from the lung tissue, was chosen as a control cell to monitor the effect of treatments on normal cells. MRC−5 was cultured in Eagle’s Minimum Essential Medium (EMEM) supplemented with 10% fetal bovine serum (FBS) and 100 U penicillin / 0.1 mg/mL streptomycin. The justification for using this cell line was because it has been reported that about 60% of people diagnosed with metastatic breast cancer have lesions in either the lungs or the bones. Triple−negative disease is more likely than other types of breast cancer to metastasize to the lungs.

The culture media were changed every two days. Cells were passaged routinely. MDA−MB−468 and MRC−5 were detached using 0.25% trypsin–EDTA (Nacalai Tesque Inc, Tokyo), and BT−20 was detached using TrypLE Select Enzyme 10x solution (Gibco, ThermoFisher Scientific: Waltham, MA, USA) and counted using a hemocytometer.

### 4.3. Measuring Antiproliferative Assay using Real−Time Cell Analyzer (RTCA)

The cell index (CI) was acquired by the RTCA iCELLigence™ system (ACEA Biosciences, Inc., San Diego, CA, USA). All monitoring was performed at 37 °C in a humidified atmosphere with regulated 5% CO_2_. E−plates (culture plates for the iCELLigence system) containing 100 µL culture medium per well were equilibrated to 30 °C, and the CI was set to zero under these conditions. For each cell type, 1 × 10^4^ cells per well were seeded into 100 µL of media in a 16−well E−plate. The cells were allowed to settle down into the E−plate at room temperature for half an hour. The cells were monitored every 30 min using the xCELLigene system for 24 h. The media was then replaced with a new 100 µL of media containing 1% of the respective drug concentrations in every well of the E−plates. The vehicle control was included, containing 1% DMSO, as well as a positive control containing 5% DMSO with media. After treatment, the E−plates were incubated and monitored every 15 min for 72 h using the xCELLigene system. Data for cell adherence were normalized at 24 h [52,53].

To determine the IC_50_, at least three concentrations of talazoparib and calcitriol were treated in the E−plate. BT−20 was treated with 100 to 200 µM talazoparib and MDA−MB−468 of a concentration from 31.25 µM to 1 mM (2x serial dilution). Calcitriol treatment concentrations were 1, 5 and 10 µM for both cell lines. The antiproliferative effect was evaluated by determining the IC_50_ of each treatment at 48 and 72 h after seeding the cells (including 24 h of seeding time) (cell index was monitored until it dropped to 50%. that time point was captured and listed in Table 1 and Table 2. Please refer to Supplementary Files S1 and S2 to find out an example of this calculation. RTCA Data Analysis Software version 2.0 was used to calculate the IC_50_ values (ACEA Biosciences, Inc.)).

IC_50_ of talazoparib and calcitriol was determined by an antiproliferative assay; it was used for the following experiments, including cell migration, apoptosis, and cell cycle analysis.

### 4.4. Cell Migration Analysis

The rate of cell migration was monitored using the real−time xCELLigence system, with fetal bovine serum (FBS) as a chemoattractant. A total of 160 µL of 10% FBS complete media was loaded with reverse pipetting skill into the lower chambers (LC) of the CIM−plate 16, and the last wells were loaded with serum−free media as a negative control. The upper chambers (UC) were assembled with the LC with a click sound according to the manufacturer’s recommendation. According to manufacturer guidelines, a total of 50 µL of serum−free media was then loaded into the UC and placed in the CO_2_ incubator for an hour for temperature equilibration to 37 °C according to manufacturer guidelines. The CIM−plate was placed in the xCELLigence system with only media, as a blank with no cells. The UC media was then replaced with 3 × 10^4^ cells per well with new 100 µL of media containing 1% of the respective drug concentrations in serum−free media. The CIM−plate was equilibrated at room temperature to let the cells settle down for half an hour. Then, the CIM−plate was placed into the xCELLigence system to monitor cell migration every 15 min for 24 h in the CO^2^ incubator. The doubling of cells is the main factor that determines the length of a migration assay. In this view, 24 h impedance measurements reflected the cell lines’ migration from the upper chamber to the lower chamber. After 24 h, the RTCA software was stopped. Data were collected, and cell index curves were analyzed to determine the cell migration rate [52].

### 4.5. Apoptosis and Cell Cycle Analysis Using Flow Cytometry

According to the manufacturer’s instructions, apoptosis was measured using the Annexin V−FITC / PI Apoptosis Detection kit (Elabscience, Houston, TX, USA). BT−20 and MDA−MB−468 cells (1 × 10^6^ cells/well) were treated for 24 and 72 h, respectively, with various concentrations of talazoparib, calcitriol and their combination in a 6−well plate (Based on the IC_50_ obtained by the RTCA software). The cells were harvested and washed with chilled PBS in a polystyrene round−bottom tube prior to suspension in 100 µL Annexin−binding buffer (ABB). Subsequently, the cells were stained with 2.5 µL of Annexin V and 2.5 µL of propidium iodine (PI) staining solution for 15 min. Staining was performed in the dark at room temperature. A total of 400 µL of ABB was then added to the stained cells prior to analysis with the FACSCanto II flow cytometer (BD Bioscience, Franklin Lakes, NJ, USA). For each measurement, at least 10,000 cells were counted.

For cell cycle analysis, BT−20 and MDA−MB−468 with a density of 1 × 10^6^ cells/mL were treated with various concentrations of talazoparib, calcitriol and their combinations for 24 h in a 6−well plate. The treated cells were harvested and washed with chilled PBS, centrifuged (1500 rcf, 7 min), fixed with 70% cold ethanol overnight at 4 °C, and then centrifuged again. Subsequently, cells were washed with PBS to remove excess ethanol and stained with 500 µL of 20 µg/mL PI solution (BD Bioscience) for 30 min. Staining was performed in the dark at room temperature. The cellular DNA contents were identified for detection of the cell cycle distribution using the FACSCanto II flow cytometer (BD Bioscience) installed with ModFit LT (Verity Software House). At least 10,000 events were counted for each sample.

### 4.6. Statistical Analysis

All data were statistically analyzed using SPSS version 22. Data are shown as the mean ± standard deviation (SD) of three independent experiments. Multiple comparisons of the cell apoptosis and cell cycle assay were evaluated for statistical significance by the one−way ANOVA post−hoc test (Tukey), and data significance levels are shown as *p* < 0.05.

## 5. Conclusions

In this study talazoparib and calcitriol combination showed a proliferation inhibitory effect on two TNBC cell lines with BRCA wild−type and BRCA1 allelic loss. The combined therapy also has affected the cell migration, apoptosis, and necrosis rates in these cell lines. BT−20 was more sensitive to talazoparib. The combination of talazoparib and calcitriol could be useful as a new formulation to treat TNBC.

An animal study should be carefully planned to confirm the results of this in vitro study. Future studies should also focus on improving and optimizing combined treatments for TNBC patients by determining the best duration, frequency, and concentration, as well as identifying and verifying biomarkers for patient selection and stratification.

These data strongly suggest future clinical investigation of a combination of PARP inhibitors and calcitriol, which has the potential to dramatically improve the efficacy of innovative targeted therapy for TNBC patients with varying BRCA1/2 status.

## Figures and Tables

**Table 1:** The IC_50_ concentrations on the BT−20 cell line and the relevant time points.

Treatments	IC50 (µM)	Time (h)
Talazoparib	91.6	61
Calcitriol Combination	10 both IC_50_	28 30

**Table 2:** The IC_50_ concentration of talazoparib and calcitriol in the MDA−MB−468 cell line and the relevant time points.

Treatments	IC50 (µM)	Time (h)
Talazoparib	1000	69
Calcitriol Combination	10 both IC_50_	50 34

## Data Availability

Data is contained within the article and supplementary material.

## Supplementary Materials

The following supporting information can be downloaded at: https://www.mdpi.com/article/10.3390/ph15091075/s1, File S1: Antiproliferative effect of talazoparib and calcitriol on BT−20.; File S2: Antiproliferative effect of talazoparib and calcitriol on MDA−MB−468 cells; File S3: Treatments effect in MRC−5 using BT−20 concentrations; File S4: Treatments effect in MRC−5 using MDA−MB−468 concentration; File S5: BT−20 cell migration profiles; File S6: MDA−MB−468 cell migration profiles; File S7: BT−20 Apoptosis; File S8: MDA−MB−468 Apoptosis; File S9: BT−20 cell cycle; File S10: MDA−MB−468 cell cycle; Table S1: Comparison of Significant Different between Talazoparib, Calcitriol and their combination in Cell Migration Assay using Anova SPSS–(BT−20); Table S2: Comparison of Significant Different between Talazoparib, Calcitriol and their combination in Cell Migration Assay using Anova SPSS–(MDA−MB−468); Table S3: Comparison of Significant Different between Talazoparib, Calcitriol and their combination in Annexin V/PI Flow Cytometry using Anova SPSS–(BT−20); Table S4: Comparison of Significant Different between Talazoparib, Calcitriol and their combination in Annexin V/PI Flow Cytometry using Anova SPSS–(MDA−MB−468); Table S5: Comparison of Significant Different between Talazoparib, Calcitriol and their combination in Cell Cycle Analysis using Anova SPSS–(BT−20); Table S6: Comparison of Significant Different between Talazoparib, Calcitriol and their combination in Cell Cycle Analysis using Anova SPSS–(MDA−MB−468).
