# Supplementary material for: Combination of Talazoparib and Calcitriol Enhanced Anticancer Effect in Triple−Negative Breast Cancer Cell Lines
Source: Pharmaceuticals (Basel). 2022 Aug 29;15(9):1075. doi: 10.3390/ph15091075 (PMC9504984; doi:10.3390/ph15091075)
Supplement: Supplementary file 1 [file pharmaceuticals-15-01075-s001.zip › Table supplementary.pdf]

Table S1

Comparison of Significant Different between Talazoparib, Calcitriol and their combination in Cell Migration Assay using Anova SPSS– (BT-20)

Cell Migration

	Control	Talazoparib	Calcitriol	T+C
Control		0.132	0.347	<0.001*
Talazoparib	0.132		0.007*	<0.001*
Calcitriol	0.347	0.007*		<0.001*
T+C	<0.001*	<0.001*	<0.001*	

* $p < 0.05$

Table S2

Comparison of Significant Different between Talazoparib, Calcitriol and their combination in Cell Migration Assay using Anova SPSS– (MDA-MB-468)

Cell Migration

	Control	Talazoparib	Calcitriol	T+C
Control		<0.001*	<0.001*	<0.001*
Talazoparib	<0.001*		0.504	0.504
Calcitriol	<0.001*	0.504		1.00
T+C	<0.001*	0.504	1.00	

* $p < 0.05$

Table S3

Comparison of Significant Different between Talazoparib, Calcitriol and their combination in Annexin V/PI Flow Cytometry using Anova SPSS– (BT-20)

Viable

	Control	Talazoparib	Calcitriol	T+C
Control		0.972	<0.001*	<0.001*
Talazoparib	0.972		<0.001*	<0.001*
Calcitriol	<0.001*	<0.001*		<0.001*
T+C	<0.001*	<0.001*	<0.001*	

Early Apoptosis

	Control	Talazoparib	Calcitriol	T+C
Control		0.99	1.00	0.451
Talazoparib	0.99		1.00	0.364
Calcitriol	1.00	1.00		0.508
T+C	0.451	0.364	0.508	

Late Apoptosis

	Control	Talazoparib	Calcitriol	T+C
Control		1.00	<0.001*	<0.001*
Talazoparib	1.00		<0.001*	0.997
Calcitriol	<0.001*	<0.001*		<0.001*
T+C	<0.001*	0.997	<0.001*	

Necrosis

	Control	Talazoparib	Calcitriol	T+C
Control		0.569	<0.001*	0.976
Talazoparib	0.569		<0.001*	0.921
Calcitriol	<0.001*	<0.001*		<0.001*
T+C	0.976	0.921	<0.001*	

* $p < 0.05$

Table S4

Comparison of Significant Different between Talazoparib, Calcitriol and their combination in Annexin V/PI Flow Cytometry using Anova SPSS – (MDA-MB-468)

Viable

	Control	Talazoparib	Calcitriol	T+C
Control		<0.001*	0.094	<0.001*
Talazoparib	<0.001*		<0.001*	0.003*
Calcitriol	0.094	<0.001*		<0.001*
T+C	<0.001*	0.003*	<0.001*	

Early Apoptosis

	Control	Talazoparib	Calcitriol	T+C
Control		<0.001*	0.766	<0.001*
Talazoparib	<0.001*		<0.001*	0.378
Calcitriol	0.766	<0.001*		<0.001*
T+C	<0.001*	0.378	0.000005*	

Late Apoptosis

	Control	Talazoparib	Calcitriol	T+C
Control		0.003*	0.826	<0.001*
Talazoparib	0.003*		0.014*	0.172
Calcitriol	0.826	0.014*		0.001*
T+C	<0.001*	0.172	0.001*	

Necrosis

	Control	Talazoparib	Calcitriol	T+C
Control		0.015*	0.998	0.027*
Talazoparib	0.015*		0.023*	0.993
Calcitriol	0.998	0.023*		0.041*
T+C	0.027*	0.993	0.041*	

* $p < 0.05$

Table S5

Comparison of Significant Different between Talazoparib, Calcitriol and their combination in Cell Cycle Analysis using Anova SPSS– (BT-20)

G0/G1 phase

	Control	Talazoparib	Calcitriol	T+C
Control		<0.001*	<0.001*	<0.001*
Talazoparib	<0.001*		<0.001*	0.005*
Calcitriol	<0.001*	<0.001*		<0.001*
T+C	<0.001*	0.005*	<0.001*	

S phase

	Control	Talazoparib	Calcitriol	T+C
Control		<0.001*	<0.001*	<0.001*
Talazoparib	<0.001*		<0.001*	0.073
Calcitriol	<0.001*	<0.001*		<0.001*
T+C	<0.001*	0.073	<0.001*	

G2/M phase

	Control	Talazoparib	Calcitriol	T+C
Control		<0.001*	<0.001*	<0.001*
Talazoparib	<0.001*		<0.001*	0.004*
Calcitriol	<0.001*	<0.001*		<0.001*
T+C	<0.001*	0.004*	<0.001*	

* $p < 0.05$

Table S6

Comparison of Significant Different between Talazoparib, Calcitriol and their combination in Cell Cycle Analysis using Anova SPSS– (MDA-MB-468)

G0/G1 phase

	Control	Talazoparib	Calcitriol	T+C
Control		<0.001*	0.004*	<0.001*
Talazoparib	<0.001*		<0.001*	0.078
Calcitriol	0.004*	<0.001*		0.007*
T+C	<0.001*	0.078	0.007*	

S phase

	Control	Talazoparib	Calcitriol	T+C
Control		0.428	0.998	0.998
Talazoparib	0.428		0.493	0.491
Calcitriol	0.998	0.493		1.00
T+C	0.998	0.491	1.00	

G2/M phase

	Control	Talazoparib	Calcitriol	T+C
Control		0.176	0.243	0.106
Talazoparib	0.176		0.006*	0.954
Calcitriol	0.243	0.006*		0.005*
T+C	0.106	0.954	0.005*	

* $p < 0.05$
